# Anspruch und Wert von interdisziplinärer Kommunikation und Konsiliarbefundung

**DOI:** 10.1007/s00117-022-01113-4

**Published:** 2023-01-26

**Authors:** Benjamin Sigl, Andreas G. Schreyer, Markus Henkel, Christian Herold

**Affiliations:** 1 Universitätsklinik für Radiologie und Nuklearmedizin, Medizinische Universität Wien, Währinger Gürtel 18–20, 1090 Wien, Österreich; 2 Institut für Diagnostische und Interventionelle Radiologie, Universitätsklinikum Brandenburg an der Havel, Medizinische Hochschule Theodor Fontane, Brandenburg a. d. Havel, Deutschland; 3 Berufsverband Deutscher Radiologen, München, Deutschland

**Keywords:** Diagnostische Radiologie, Interdisziplinarität, Board, Zweitmeinung, Spezialisten, Diagnostic radiology, Interdisciplinarity, Board meeting, Second opinion, Specialists

## Abstract

Interdisziplinäre Kommunikation und Konsiliarbefundungen nehmen einen relevanten Anteil der radiologischen Tätigkeit ein. Sie sind essenziell für eine qualitativ hochwertige und flächendeckende medizinische Versorgung. Es gibt verschiedene Modalitäten der interdisziplinären Kommunikation mit je eigenen Vor- und Nachteilen. Dieser Artikel informiert über infrastrukturelle und personelle Anforderungen sowie wichtige medikolegale Aspekte von Zweitbefundungen und interdisziplinären Boards. Zudem wird die eklatante Diskrepanz zwischen dem damit verbundenen Aufwand für ein Institut und der unzureichenden Abbildung in den Abrechnungssystemen offenbart.

## Abstract (en)

Interdisciplinary communication and consultation take up a relevant part of the radiological workload. They are essential for high-quality and ubiquitous medical care. There are different modalities of interdisciplinary communication, each with its own advantages and disadvantages. This article provides information on requirements regarding infrastructure and personnel as well as important medicolegal aspects of second opinion reports and interdisciplinary boards. It also reveals the striking discrepancy between the effort required by an institute and the inadequate reflection regarding remuneration in the billing systems.

## Kommunikation als Teil des radiologischen Alltags

Jeder radiologische Befundreport ist verschriftlichte Kommunikation, sowohl mit dem Patienten als auch mit den an seiner Behandlung beteiligten Personen. Doch im klinischen Alltag ist diese Art der Kommunikation allein oftmals nicht ausreichend. Daher haben sich verschiedene Wege der interdisziplinären Kommunikation etabliert, welche häufig nicht nur den initial Befundenden, sondern auch radiologische Zweitbefundungen beinhalten. Steter technischer und diagnostischer Fortschritt machen es dem einzelnen Radiologen schier unmöglich, in allen Gebieten Wissen auf Expertenniveau zu akquirieren und zu halten. Gerade mit zunehmender Spezialisierung der Medizin im Allgemeinen und der Radiologie im Speziellen sowie den immer komplexer werdenden Diagnose- und Therapiemöglichkeiten ist auch seitens der Radiologie eine aktive Kommunikation mit anderen Fachdisziplinen integraler Bestandteil unserer Arbeit. Dies gilt insbesondere in Kliniken, aber auch in radiologischen Praxen [1].

Durch fortschreitende Subspezialisierung auf diversen radiologischen Teilgebieten und zunehmend zentrenorientierter Medizin einerseits sowie durch Entwicklungen in Digitalisierung und Vernetzung innerhalb der Radiologie andererseits entstehen neue Möglichkeiten, aber auch der dringende Bedarf an interdisziplinären Befundbesprechungen und Konsiliarbefundungen durch Experten. Demgegenüber steht die sich zuspitzende Problematik, gerade in strukturschwachen Regionen einen Zugang zu radiologischen Untersuchungen für eine flächendeckende medizinische Versorgung zu gewährleisten.

Eine mögliche Lösung wird regelmäßig in der Einführung von teleradiologischer Befundung gesehen, insbesondere außerhalb der regulären Arbeitszeiten und in versorgungsschwachen Regionen. Der generelle Trend – nicht zuletzt auch resultierend aus der Corona-Pandemie – hin zu mehr Flexibilität im Arbeitsumfeld und der Möglichkeit von Home Office wird auch in der Radiologie zunehmend gefordert und angeboten [2]. Wie kaum einem anderen Fachgebiet der Medizin ermöglichen die technischen Errungenschaften der letzten Jahre eine Umsetzung der Distanzbefundung. Doch wie lässt sich diese mit interdisziplinärer *Vor-Ort*-Kommunikation vereinen? Welcher Anspruch muss an die interdisziplinäre Zusammenarbeit beispielsweise in Tumorboards gestellt werden, und was sind die rechtlichen Grundlagen einer solchen Zweitmeinung? Wer haftet im Schadensfall? Und wer stemmt den Mehrbedarf an Ressourcen? Diesen Fragen wird im Folgenden nachgegangen.

## Formen der interdisziplinären Kommunikation

Wohl die meisten Radiologen werden sich schon einmal gewünscht haben, den Pager abzudrehen, das Telefon auszuschalten und die Tür zu verschließen. Doch werden radiologische Zweitmeinungen immer stärker von Kollegen anderer Fachdisziplinen gefordert. Und mehr denn je besteht auch die Notwendigkeit von radiologischer Expertise in diversen Boards, in der Beratung von Kollegen und in der Zweitbefundung, gerade bei komplexen und seltenen Erkrankungen. Die Kommunikation nimmt dabei eine Schlüsselrolle ein und kann ein breites Spektrum an Modalitäten aufweisen.

Prinzipiell kann zunächst zwischen intra- und extramuraler Kommunikation differenziert werden, wobei gerade in spezialisierten Zentren der intramurale Part überwiegt. Dabei nehmen Anfragen via Telefon oder *zwischen Tür und Angel* einen wesentlichen Teil ein. Diese Art der Anfragen stellen eine besondere Herausforderung bezogen auf Dokumentation und Nachvollziehbarkeit dar. Aber auch die zeitlichen Kapazitäten und die (häufig dem Radiologen nicht vollständig) zur Verfügung gestellten Informationen – beispielsweise Patientenakten, Vorbefunde oder schriftliche Befunde im Falle auswärtiger Untersuchungen – stehen einer optimalen Befundauskunft entgegen [3].

In der Regel ist die Aufarbeitung im Rahmen von schriftlichen Konsilanfragen und Boards vollständiger und lässt dem Radiologen mehr Zeit, sich mit dem teils ausführlichen Bildmaterial zu befassen [4]. Gerade im Rahmen strukturierter Boards ist eine Dokumentation grundsätzlich vorgesehen, allerdings erfolgt eine Freigabe des entsprechenden Dokuments nicht immer explizit auch radiologischerseits. Initialer Befunder und Boardteilnehmer/Begutachter sind dabei oft nicht dieselbe Person, sodass es sich damit um eine Zweitmeinung handelt.

Zweitmeinungen können die Therapieentscheidung relevant abändern. Untersuchungen legen beispielsweise nahe, dass die Anwesenheit eines Radiologen in spezialisierten Boards die Therapie in etwa einem Drittel der Fälle abweichend beeinflusst [4, 5]. Damit wird der Aspekt der Qualitätssicherung durch eine Zweitmeinung besonders innerhalb von Point-of-care-Zentren offenbar. Dieses Vorteils bedient man sich beispielsweise auch im Rahmen des Mammographie-Screenings, bei dem eine Zweitbefundung per se vorgesehen ist, um ein entsprechendes Niveau zu sichern. Eine zusätzliche Zweitbefundung von Untersuchungen durch Experten ihres Gebiets wird zudem teils auch außerhalb des Abrechnungssystems angeboten.

## Zweitbefundung in einer „idealen Radiologie“

In der modernen Radiologie nehmen Anfragen zu Zweitbefundungen bzw. zur Abgabe von Zweitmeinungen kontinuierlich zu. Dies ist zum einen durch die stete Zunahme von angeforderten Röntgenaufnahmen, zum anderen durch die zunehmende Auslagerung von Röntgenuntersuchung, Computertomographien (CT) und Magnetresonanztomographien (MRT) aus dem intramuralen Bereich in die Niederlassung und letztendlich auch durch die zunehmende Subspezialisierung mit teleradiologischen Konsultationen bedingt. Im intramuralen Bereich führt das mittlerweile regelhafte Einbringen von Patientinnen und Patienten mit komplexen Erkrankungen in interdisziplinäre Fallkonferenzen und Tumorboards zu einer drastischen Zunahme von Anforderungen an uns Radiologen, zusätzlich zu einer bereits erfolgten Befundung eine individuelle Meinung im Rahmen ebendieser Fallkonferenzen und Tumorboards abzugeben.

Diese Konferenzen stellen eine wichtige Schnittstelle zwischen diagnostischen Disziplinen und bettenführenden Kliniken/Stationen dar und bedeuten eine logistische wie auch personelle Investition in diese wichtigen Interaktionsinstrumente sowie nicht zuletzt eine Investition in die Zukunft unseres eigenen Faches. Die Konsequenz daraus ist jedenfalls eine stete Zunahme der inhaltlichen aber auch zeitlichen Belastung, welche in den allermeisten Fällen nicht durch zusätzliche Personalzuteilungen an radiologische Kliniken begleitet wird. So finden beispielsweise an der Universitätsklinik für Radiologie und Nuklearmedizin Wien monatlich ca. 540 derartige Fallkonferenzen und Tumorboards statt, ohne dass eine entsprechende Personalvermehrung diese massive Mehrleistung begleitet hätte. Daraus ergibt sich, dass zur Bewältigung der enormen Anforderungen das organisatorische Umfeld optimiert werden muss. Dies bedeutet eine Investition in die räumliche, apparative und technische Infrastruktur, in die personelle Ausstattung sowie letztendlich auch in die Dokumentation im Radiologie- und Krankenhausinformationssystem (KIS).

Beginnen wir mit der optimierten räumlichen Infrastruktur als Grundlage für die erfolgreiche Abhaltung von interdisziplinären Fallkonferenzen und Tumorboards. Aufgrund der Zahl der Teilnehmenden an derartigen interdisziplinären Konferenzen, die in den allermeisten Fällen zwischen 5 und 15 beträgt, sind genügend große Räumlichkeiten mit guter Belüftung ein Muss. Diese enthalten im Idealfall große Schirme, die als Hyperwall in die Raumarchitektur integriert sind und über das KIS, RIS (radiologisches Informationssystem) und das PACS („picture archiving and communication system“) ansteuerbar sind. Dadurch können sämtliche Patientendaten für alle Teilnehmenden sichtbar kommuniziert werden. Modernste Workstations und eine optimierte RIS-Struktur sind eine Grundvoraussetzung.

Um die bis zu zwei Stunden und mitunter auch länger dauernden Fallkonferenzen zu bewältigen, benötigen die Teilnehmenden ergonomische Bestuhlungen, genügend große Tische mit integrierten Netzauslässen bzw. Steckdosen zur Stromversorgung für die mitgebrachten Laptops [6]. Da während der Pandemie die überwiegende Mehrzahl dieser Konferenzen als Onlinemeetings stattgefunden haben, ist zumeist eine optimierte Infrastruktur zur Übertragung der Meetings vorhanden. Eine entsprechende Ausstattung mit Kameras und Mikrofonen ist demnach für das Abhalten von Hybrid-Meetings, also einer Kombination aus Präsenz- und Onlineteilnahme, notwendig. Dies nicht zuletzt deshalb, da aufgrund der zunehmenden Integration der Versorgungsstränge des intra- und extramuralen Bereichs eine immer größere Anzahl von externen bzw. extramuralen Spezialisten an den Tumorboards und Fallkonferenzen teilnimmt.

Ebenso ist es notwendig, den Gesamtprozess bzw. Workflow zu optimieren, um reibungslose Abläufe zu garantieren. Die Übermittlung von Worklisten mit zu besprechenden Patienten ist Grundvoraussetzung für eine zeitsparende Bearbeitung und Gesamtorganisation. Extramurale Untersuchungen sind ausnahmslos vor Beginn der jeweiligen Fallkonferenz in das PACS einzuspielen, um das zeitvernichtende Warten auf sich öffnende Dateien bzw. die regelmäßig auftretenden technischen Schwierigkeiten aufgrund inkompatibler Datensysteme zu verhindern [7]. Dies hindert klinische Kolleginnen und Kollegen anderer Fächer häufig nicht daran, trotzdem derartige „On-site“-Einbringungen zu versuchen – es hängt also von der Disziplin und Standhaftigkeit des radiologischen Moderators ab, derartige Versuche zu unterbinden.

Wie in diesem Artikel an anderer Stelle ausgeführt, ist die gemeinsame Beibringung von externen Bildern mitsamt deren Befunden unerlässlich. Dies erst ermöglicht die grundsätzlich bestehende Verpflichtung, die Zweitmeinung durch den Radiologen beispielsweise im Tumorboard mit einem externen Originalbefund abzugleichen bzw. zu etwaigen Diskrepanzen Stellung zu nehmen. Auch diesem Prozessschritt muss in der Dokumentation Rechnung getragen werden. Letztere erfordert entweder einen Tumorboard-Administrator oder eine technische Lösung, die beispielsweise mithilfe eines Spracherkennungssystems ermöglicht, direkt im HIS oder RIS zu dokumentieren.

Letztlich sind Kompetenz und Kommunikationsfähigkeit der in den Konferenzen anwesenden Radiologinnen und Radiologen von allerhöchster Wichtigkeit. Aus jahrelanger Erfahrung bestätigt sich, dass eine hohe fachliche Kompetenz nicht nur die Akzeptanz der getätigten Aussagen massiv erhöht, sondern auch hilft, beträchtlich Zeit einzusparen. Ebenso sind die Kommunikationsfähigkeit bzw. die Fähigkeit, allgemein verständliche Aussagen zu treffen, komplexe Diagnosen zu vermitteln und allfällige Diskrepanzen zu erklären, von immenser Wichtigkeit für den Erfolg der Gesamtkonferenz.

Abschließend darf nicht vergessen werden, auf die personellen Erfordernisse derartiger, zum Teil auch gesetzlich geforderter Konferenzen einzugehen. Wenn man davon ausgeht, dass ein Tumorboard oder eine interdisziplinäre Fallkonferenz durchschnittlich eine Stunde dauert und mindestens eine halbe Stunde für die Vorbereitung und nochmals ca. 30 min für eine adäquate Nachbereitung in Anspruch nimmt, ist der entsprechende Personalbedarf leicht auszurechnen. Bei 20 derartigen Konferenzen pro Woche ergibt sich daher ein Personalbedarf von mindestens einem Vollzeitäquivalent. Wenn man Urlaubszeiten sowie kongress- und erkrankungsbedingte Abwesenheit berücksichtigt, sind zwischen 1,3 und 1,5 Vollzeitäquivalente zu veranschlagen. Die Zuteilung dieser Personalressourcen stößt in zahlreichen Institutionen auf große Schwierigkeiten, vielfach auch deshalb, da diese Konferenzen in den Leistungskatalogen nicht abgebildet und daher auch nicht remuneriert werden [8].

## Rechtliche Aspekte der radiologischen Zweitmeinung

Da die rechtlichen Aspekte im deutschsprachigen Raum (Deutschland, Österreich, Schweiz) im Prinzip relativ ähnlich sind, wollen wir die entsprechenden Referenzen aus dem Strahlenschutzgesetz (StrlSchG) aus Deutschland anführen und nicht gesondert z. B. auf die Rechtsvorschrift für Medizinische Strahlenschutzverordnung (MedStrSchV) in Österreich eingehen.

Eine wichtige juristische Voraussetzung zur Erstellung einer Zweitmeinung ist die Tatsache, dass es radiologisch zunächst eine *Erstmeinung* geben muss – dies fordert bereits der strahlenschutzrechtliche Grundsatz der Einheit von Bild und Befund (Strahlenschutzgesetz [StrlSchG] § 85). Daraus ergibt sich zwangsläufig die primäre Forderung, dass zur Erstellung einer Zweitmeinung der radiologische Befund des primären Bilderstellers schriftlich vorliegen sollte [9].

Entscheidende Gründe dafür liegen in der Tatsache, dass dem Zweitbefundenden ggf. wichtige klinische Primärangaben, technische Parameter der Untersuchung und die Möglichkeit eines Patientengespräches nicht mehr möglich sind.

Bei vorliegendem Zeitdruck in einer Notfallsituation kann auf diese Vorbefunde verzichtet werden und diese ggf. nach Beendigung der Notfallsituation nachgeholt werden. In einer elektiven Situation sollte jedoch auf das Vorliegen der primären Befunddokumentation bestanden werden.

Als Radiologe muss man sich bei der konsiliarischen Bewertung als Zweitmeinung, wie sie nicht nur im elektiven Umfeld, sondern auch in Röntgenbesprechungen und Tumorboards als *Ad-hoc-*Situation täglich in der Routine vorkommt, im Klaren sein, dass die geäußerte Meinung als diagnostische Leistung eine eigenständige radiologische Arbeit darstellt, für die man, auch juristisch, allein die Verantwortung trägt. Dies bedeutet u. a., dass man sich nicht einfach auf den Vorbefund verlassen darf, sondern als Konsil eine vollständige und eigenständige Befundung leisten muss. Dies leitet sich bei radiologischer Konsiliartätigkeit aus § 630a BGB in Bezug auf die Sorgfaltspflicht eines Konsiliararztes ab. Aus diesem Grund wird von Juristen, wie so häufig in der Medizin, eine sorgfältige schriftliche bzw. elektronische Dokumentation empfohlen. Fast ebenso häufig ist diese Forderung zuweilen im extrem dicht gepackten klinischen Alltag bei den vielen informellen telefonischen Anfragen bzw. der persönlichen kollegialen Zweitbefundung *zwischen Tür und Angel* nicht möglich, in vielen Fällen jedoch dann auch nicht nötig. Juristisch sollten wir jedoch darauf achten, dass gerade bei kritischen Entscheidungssituationen, wie etwa Tumorboards, oder kritischen Befundsituationen, in denen z. B. dem Erstbefundenden in relevanten Punkten widersprochen wird, unbedingt eine ausreichende schriftliche Dokumentation vorliegt.

Interessant erscheint gerade bei Widersprüchen zwischen Erst- und Zweitbefundung die Frage, welcher Befund denn nun der korrekte, der klinisch bindende Befund ist. Juristisch handelt es sich zunächst um die fachgleiche Leistung von zwei radiologischen Fachärzten. Der Radiologe in der Zweitbefundung hat, wie oben erwähnt, die Bildgebung vollständig eigenständig zu evaluieren und kann in seinem Befund inhaltlich ggf. auf den Primärbefund eingehen. Die Meinung des Zweitbefundenden ist also, auch wenn divergierend, zunächst ebenso gleichwertig als gültiger Befund zu betrachten. Eine klinische bzw. medikolegale Bevorzugung, also eine Privilegierung eines der beiden Befunde kann es lediglich geben, wenn der Erstbefund aus einem unterschiedlichen Fachgebiet stammt (z. B. wenn ein Orthopäde die Erstbefundung angefertigt hat). Ansonsten stehen sich die divergierende Erst- und Zweitbefundung als Meinung des jeweils befundenden Radiologen, also als zwei subjektive *Wahrheiten* gegenüber. Die Entscheidung, welcher der beiden Befunde bzw. welche der beiden Meinungen vom patientenführenden Arzt als richtig und damit als bindend aufgefasst wird, muss der behandelnde Arzt letztendlich selbst treffen. Hier ist es jedoch bei einem Befundkonflikt sehr ratsam, eine schriftliche bzw. elektronische Dokumentation der abwägenden Meinungsbildung im RIS bzw. KIS für potenzielle künftige juristische Konflikte zu hinterlegen.

Es gibt also von Seiten der Radiologie bei zwei divergierenden Befunden rechtlich zunächst keinen bindenden, *besseren* Befund – die Entscheidung, welcher diagnostischen Meinung gefolgt wird, wird vom behandelnden Arzt getroffen. Auch eine radiologische Schwerpunktbezeichnung (z. B. in Deutschland Schwerpunkt Neuroradiologe bzw. Kinder- und Jugend-Radiologie) hat zunächst keinen Einfluss auf die rechtliche *Wertigkeit* des Befundes, zumal sie lediglich eine Spezialisierung innerhalb und aufbauend auf der Facharztausbildung der Radiologie darstellt.

Zusammenfassend muss noch einmal betont werden, dass jede konsiliarische Zweitmeinung eine eigene persönliche Leistung eines Radiologen darstellt, für welche die übliche Sorgfaltspflicht in der Medizin und damit auch persönliche Verantwortung gilt. Als eigene Leistung kann also nicht vollständig auf den Erstbefund vertraut werden, der jedoch zur Bildung der Zweitbefundung vorliegen sollte.

## Anforderungen und Mindeststandards konsiliarischer radiologischer Befunde

Basierend auf den vorgenannten juristischen Grundlagen sind die Anforderungen für eine elektive erstellte radiologische Zweitmeinung unter Berücksichtigung der geforderten Sorgfaltspflicht einer ärztlichen Leistung zunächst das Vorliegen des Erstbefunds (Einheit von Bild und Befund – Strahlenschutzgesetz). Da auch eine Zweitbefundung eine eigenständige ärztliche diagnostische Leistung darstellt, muss eine vollständige Würdigung aller Bilddaten, ggf. sogar unter Berücksichtigung von Voraufnahmen, erfolgen. Sollten Voruntersuchungen bzw. primäre radiologische Befunde oder mangelnde klinische Informationen die Beurteilung erschweren oder einschränken, müssen diese Punkte in der schriftlichen Zweitmeinung erwähnt und dokumentiert werden.

## Fazit für die Praxis

- Zunehmende Spezialisierung, zentren- und patientenorientierte Medizin sowie fortschreitende diagnostische und therapeutische Möglichkeiten machen die Notwendigkeit interdisziplinärer Kommunikation offenkundig und begründen die steigende Nachfrage.
- Als integraler Bestandteil sind spezialisierte Radiologen in interdisziplinären Boards unabdingbar und sorgen in etwa einem Drittel der Fälle für relevante Therapieänderungen.
- Überregional organisierte Boards im Onlineformat können radiologische Expertise in strukturschwachen Regionen sichern.
- Beteiligung an interdisziolinären Boards und Konsiliarbefunde sind folglich wichtig und sollten – natürlich in einem vernünftigen Rahmen – angeboten werden. Sie treiben Expertise, Relevanz für das Fach und nicht zuletzt unsere Identitätsbildung voran.
- Auch wenn die Realität oft weit von den Vorstellungen einer „idealen Radiologie“ entfernt ist, so gibt es für den Umgang mit Zweitmeinungen einen rechtlichen und ethischen Mindestanspruch, für dessen Einhaltung Ressourcen (u. a. Arbeitskraft, IT, Räumlichkeiten) notwendig sind. Diese werden jedoch in den aktuellen Abrechnungssystemen kaum abgebildet und bedeuten in der praktischen Umsetzung eine relevante Mehrbelastung für die Institutionen.
- Es ist ein Umdenken in der Politik notwendig, um diesen essentiellen Bestandteil der interdisziplinären Medizin zu sichern.
